# Multiple Active Compounds from *Viscum album L*. Synergistically Converge to Promote Apoptosis in Ewing Sarcoma

**DOI:** 10.1371/journal.pone.0159749

**Published:** 2016-09-02

**Authors:** Monika Twardziok, Susann Kleinsimon, Jana Rolff, Sebastian Jäger, Angelika Eggert, Georg Seifert, Catharina I. Delebinski

**Affiliations:** 1 Department of Pediatric Oncology/Hematology, Otto Heubner Centre for Pediatric and Adolescent Medicine (OHC), Charité, Universitätsmedizin Berlin, Germany; 2 Institute of Pharmacy, Department of Biology, Chemistry, Pharmacy, Freie Universität, Berlin, Germany; 3 EPO GmbH, Experimental Pharmacology & Oncology, Berlin, Germany; 4 Birken AG, Niefern-Öschelbronn, Germany; Johns Hopkins University, UNITED STATES

## Abstract

Ewing sarcoma is the second most common bone cancer in children and adolescents, with poor prognosis and outcome in ~70% of initial diagnoses and 10–15% of relapses. Hydrophobic triterpene acids and hydrophilic lectins and viscotoxins from European mistletoe (*Viscum album* L.) demonstrate anticancer properties, but have not yet been investigated for Ewing sarcoma. Commercial *Viscum album* L. extracts are aqueous, excluding the insoluble triterpenes. We recreated a total mistletoe effect by combining an aqueous extract (viscum) and a triterpene extract (TT) solubilized with cyclodextrins. Ewing sarcoma cells were treated with viscum, TT and viscumTT *in vitro*, *ex vivo* and *in vivo*. *In vitro* and *ex* vivo treatment of Ewing sarcoma cells with viscum inhibited proliferation and induced apoptosis in a dose-dependent fashion, while viscumTT combination treatment generated a synergistic effect. Apoptosis occurred via intrinsic and extrinsic apoptotic pathways, evidenced by activation of both CASP8 and CASP9. We show that viscumTT treatment shifts the balance of apoptotic regulatory proteins towards apoptosis, mainly via CLSPN, MCL1, BIRC5 and XIAP downregulation. ViscumTT also demonstrated strong antitumor activity in a cell line- and patient-derived mouse model, and may be considered an adjuvant therapy option for pediatric patients with Ewing sarcoma.

## Introduction

Ewing sarcoma, while the second most common bone sarcoma in children and adolescents (peaking in the second decade), is rare and occurs in ~2.6 and ~2.8 per million children in the United States and Germany, respectively [1,2]. It originates from either mesenchymal stem or neuronal crest cells [3,4]. Pathogenesis results from a balanced translocation of the *EWS* gene producing fusion proteins coding for chimeric transcription factors promoting cell growth. EWS-FLI1 is the most frequent fusion protein [5,6]. Therapeutic advances in the last few years support five-year survival in 70% of Ewing sarcoma patients. Standard therapy currently combines surgery, chemotherapy and radiotherapy, but relapse tumors are often drug resistant [7]. Patient outcome for relapsed Ewing sarcoma is poor, with cure in only 10–15% of patients [8]. Stage, anatomical localization, and tumor size influence prognosis [7]. Expression of the inhibitor of apoptosis protein (IAP) family member, BIRC5 (formerly survivin), is also a poor prognostic factor for Ewing sarcoma [9,10]. BIRC5 has been suggested as an attractive target for new anticancer agents, since it is expressed in many cancers but not in differentiated normal tissues [11]. XIAP, another IAP family member, is also overexpressed in multiple cancers and is associated with drug resistance, making it another promising therapeutic target [12,13]. Combining targeted agents in current protocols might improve survival by reducing resistance development.

European mistletoe, *Viscum album* L., has been popular in anthroposophic medicine for decades. A broad range of biologically active substances have been identified in *Viscum album* L., and include viscotoxins, flavonoids, triterpene acids and mistletoe lectins [14–18]. Commercial aqueous *Viscum album* L. extracts contain the hydrophilic mistletoe lectins (ML) I-III, which are the best studied compounds from mistletoe [19,20]. MLI-III have been demonstrated to stimulate the immune system and induce apoptotic cell death in cell lines derived from head and neck squamous cell carcinomas [21] and rat glioma [22] as well as in a single alternatively treated patient with a stage IIIC colon carcinoma [23].

The hydrophobic triterpene acids, oleanolic, betulinic and ursolic acid, represent another potent group of mistletoe-derived substances, although their low solubilities exclude them from commercially available aqueous extracts [24]. Solubilizing mistletoe triterpene acids (mainly oleanolic and betulinic acids) with cyclodextrins in a buffered aqueous solution produces the triterpene extract, TT. Triterpene acids, including oleanolic acid, its derivatives and betulinic acid, inhibit cell growth and induce apoptosis in cell lines derived from breast [25], ovarian [26] and nonsmall cell lung cancers [27] as well as neuroectodermal tumors *in vitro* and *ex vivo* [28]. Combination of oleanolic and ursolic acid has been reported to act synergistically against melanoma cells *in vitro* and *in vivo* [29].

We and others have already demonstrated the therapeutic effect of recombining hydrophilic and hydrophobic mistletoe constituents in the viscumTT extract for acute lymphoblastic and myeloid leukemia *in vitro* and *in vivo* [30,31] and murine melanoma *in vivo* [32]. Here, we analyzed for the first time the cytotoxic effect of viscumTT and its single extracts in Ewing sarcoma *in vitro*, *ex vivo* and *in vivo*.

## Materials and Methods

### *Viscum album* L. extracts

Viscum and TT extracts were prepared from *Viscum album* L. harvested from apple trees (*malus*) as previously described [30,31,33] by the Birken AG (Niefern-Oeschelbronn, Germany), who kindly provided lyophilized viscum and TT extracts. Intact ML-I (with A+B chain) was quantified by ELISA in viscum extract [34]. Oleanolic acid and betulinic acid quantification was performed by GC-FID [33]. Lyophilized viscum extract was reconstituted in phosphate-buffered saline (Gibco^®^ Life Technologies, Darmstadt, Germany) to a final concentration of 2μg/mL intact ML-I and <1μg/mL viscotoxins. Lyophilized TT extract (containing cyclodextrins) was reconstituted in phosphate-buffered saline to a final concentration of 4000μg/mL oleanolic and 350μg/mL betulinic acid.

### Cell culture

Human Ewing sarcoma cell lines were obtained from the German Collection of Microorganisms and Cell Cultures (DSMZ, Braunschweig, Germany). TC-71 was maintained in Iscove's Modified Dulbecco's Medium with L-glutamine (Gibco^®^ Life Technologies, Darmstadt, Germany). MHH-ES-1 was maintained RPMI 1640 base medium with L-glutamine (Gibco Lifetechnologies). Base media were supplemented with 10% heat-inactivated fetal calf serum (Biochrom, Berlin, Germany), 100U/mL penicillin and 100μg/mL streptomycin (Biochrom). For assays, 2x10^5^ TC-71 cells and 4x10^5^ MHH-ES-1 cells were seeded onto 6-well plates (half the cell number onto 12-well microtiter plates), cultured 24h to allow cell attachment and treated 24h with *Viscum album* L. extracts added to culture media.

### *Ex vivo* cultured Ewing sarcoma primary cells

A tumor sample was obtained as treatment 'residue' from a 15-year-old girl with Ewing sarcoma during routine surgical resection, and was not explicitly collected for this research. Diagnosis was confirmed by histopathology. The sample was dissected into smaller pieces immediately after surgical excision, then cultured as a primary explant in RPMI 1640 base medium with L-glutamine supplemented with 20% heat-inactivated fetal calf serum and 1% penicillin/streptomycin solution (Biochrom) to obtain dissociated monolayer culture outgrowth from the explant. Confluent *ex vivo* cell cultures were treated within 4 trypsinized passages. CD99 expression confirmed *ex vivo* cultures to be Ewing sarcoma using immunocytochemistry and flow cytometry with FITC-labeled anti-CD99 antibody (#561986, BD Biosciences, Franklin Lakes, NJ, USA) versus the isotype control antibody (#555573). FISH confirmed the *EWS-ETS* translocation in the *ex vivo* culture. Cells were seeded into 12-well microtiter plates at 1.3x10^5^/well, cultured 24h to allow cell attachment and treated 24h with *Viscum album* L. extracts added to culture media. Written informed consent was obtained from the patient's parents and/or legal guardians in accordance with the Declaration of Helsinki, approved by the local ethics committee of Charité—Universitätsmedizin Berlin.

### Ewing sarcoma xenografts and experimental procedures

Eight-week-old female NOD/SCID IL2rγ null mice and 6-8-weeks-old female NMRI-nu/nu mice were obtained from Taconic or in-house breeding, housed in a pathogen-free facility under pathogen-free conditions and fed autoclaved standard diet (Sniff, Soest, Germany) with acidified drinking water *ad libitum*.

A) TC-71 cells (1x10^6^) were subcutaneously injected in saline into the left flank of 8 NOD/SCID IL2rγ null mice per treatment or control group. Intratumoral treatment with viscum, TT, viscumTT or cyclodextrins alone (control group) began on day 12 when tumors were palpable. Intravenous treatment with viscumTT or cyclodextrins began on day 3 after tumor cell injection. Mice were treated with increasing extract concentrations due to better tolerability: 40/50/60mg/kg oleanolic acid (TT), 0.75/1.25/1.75μg/kg mistletoe lectin (viscum) or a combination thereof (viscumTT). Doses were administered every 2–3 days (Mon/Wed/Fri/Mon/Fri) in increasing concentrations and each concentration was given twice within 2–3 days due to better tolerability of the mistletoe extracts. The standard-of-care group received doxorubicin with 4 mg/kg once, intravenously on day 12.

B) Patient-derived Ewing sarcoma fragments were subcutaneously transplanted into the left flank of 5 mice per treatment or control group. Intratumoral and intravenous treatment with viscumTT, cyclodextrins alone or doxorubicin (positive control group) began on day 48 when tumors were palpable. Mice were treated with increasing extract concentrations of 40/60/80mg/kg oleanolic acid and 0.75/1.25/1.75μg/kg mistletoe lectin in combination (viscumTT, low dose) or 50/70/90mg/kg oleanolic acid and 1.25/1.75/2.25μg/kg mistletoe lectin in combination (viscumTT, high dose). Doses were administered every 2–3 days (Wed/Fri/Mon/Wed/Fri/Mon) in increasing concentrations and each concentration was given twice within 2–3 days. One positive control group received doxorubicin, 4 mg/kg, intravenously on day 48 and day 55.

Body weight and tumor volume was measured twice weekly, and mice were daily monitored for health and symptoms of toxicity. Animals were sacrificed by cervical dislocation at the end of the experiment or if mice were moribund (tumor volume >1.2cm^3^ or >10% body weight lost). Animal experiments were performed in accordance with German legislation on the care and use of laboratory animals and in accordance with the United Kingdom Coordinating Committee on Cancer Research Guidelines for the Welfare of animals in Experimental Neoplasia to minimize suffering. Approval for the study was obtained from the Regional Office for Health and Social Affairs (LaGeSo, approval G-0030/15).

### Determining cell proliferation and early cytotoxicity

Cells were counted using a CASY^®^ Cell Counter (Schaerfe System GmbH, Reutlingen, Germany). Cell proliferation was estimated from total numbers of cells in cultures started from defined cell numbers compared to control cultures. Early cytotoxicity was assessed after 2h of incubation with extracts by photometrically (490nm) monitoring lactate dehydrogenase (LDH) release into culture medium using the Cytotoxicity Detection Kit (Roche Diagnostics GmbH, Mannheim, Germany) according to manufacturer´s instructions.

### Assessing apoptotic cell death

Cells were stained with APC-conjugated annexin V (BD Biosciences) and 1 mg/mL propidium iodide (Sigma-Aldrich, München, Germany) according to manufacturer’s instructions to assess apoptosis. The mitochondrial membrane potential (ΔΨ_m_) was measured by JC-1 staining (AAT Bioquest, Sunnyvale, CA, USA) according to manufacturer’s instructions. CASP3, CASP8 and CASP9 activity were measured using the Green Caspase Staining Kit (Promokine, Heidelberg, Germany) with the nontoxic, irreversibly caspase-binding cell-permeable FITC-LEHD-FMK, FITC-IETD-FMK or FITC-DEVD-FMK, according to manufacturer’s directions. Cells stained using any of these protocols were analyzed by flow cytometry (FACS Calibur, BD Biosciences), and results were evaluated with FlowJo Software (TreeStar, Ashland, OR, USA).

### Caspase inhibitor assay

TC-71 cells were incubated with 40μM Z-VAD-FMK (R&D systems, Minneapolis, MN, USA), a pan-caspase inhibitor before treatment with viscum, TT or viscumTT extracts at ~IC50 concentrations. DMSO was added to extracts as solvent control. Apoptotic induction was determined by flow cytometry after annexin V/propidium iodide staining.

### Proteome profiler human apoptosis array and western blotting

TC-71 cells were washed twice with phosphate-buffered saline, lysed in Lysis Buffer 17 (R&D systems) containing complete Protease Inhibitor Cocktail (Roche Diagnostics GmbH) and protein concentration determined by Bradford assay (Bio-Rad, München, Germany). Lysate (300μg protein) was incubated with the Proteome Profiler Human Apoptosis Array (R&D systems) according to manufacturer´s instructions. Chemoluminescence was visualized by densitometry on a Molecular Imager ChemiDoc (Bio-Rad) to detect relative expression of apoptosis-related proteins. Expression was validated using western blotting. TC-71 and MHH-ES-1 cell lysates (30μg protein/lane) were separated on SDS-PAGE, transferred to nitrocellulose membranes (Bio-Rad system) and blocked with 5% nonfat milk in 50mM TBS with 0.05% Tween-20 (TBST) for 1h at room temperature. Blots were incubated overnight at 4°C in TBST containing 5% bovine serum albumin and primary antibody, washed 3X in TBST and incubated 1h with HRP-conjugated secondary antibodies (ant-rabbit or anti-mouse, Bio-Rad), then visualized by ECL (Thermo Fisher Scientific, Bonn, Germany) on a Molecular Imager ChemiDoc (Bio-Rad). Primary antibodies used were directed against CASP3 (#9662, Cell Signaling Technology, Danvers, MN, USA), PARP1 (#9542, Cell Signaling Technology), CLSPN (#2800, Cell Signaling Technology), BIRC5 (#2803, Cell Signaling Technology), TP53 (sc-73566, Santa Cruz Biotechnology, Santa Cruz, CA, USA), PMAIP1 (sc-56169, Santa Cruz Biotechnology) and MCL1 (sc-819, Santa Cruz Biotechnology), XIAP (#610716, BD Biosciences) and ß-actin (#A3854, Sigma-Aldrich).

### Statistics

*In vitro* experiments (except the Proteome Profiler Human Apoptosis Array) were performed in three independent experiments, for which means ±standard error are plotted in bar graphs. A Webb´s fractional product (Fp) >1 determined that the effect of viscumTT on apoptotic induction *in vitro* and *ex vivo* was synergistic, as described [30]. One-way ANOVA and Fisher´s LSD post-hoc tests were applied to determine differences between mouse xenograft treatment groups. All results with p ≤ 0.05 were considered significant.

## Results

### ViscumTT inhibits proliferation and induces apoptosis *in vitro*

**Fig 1:**
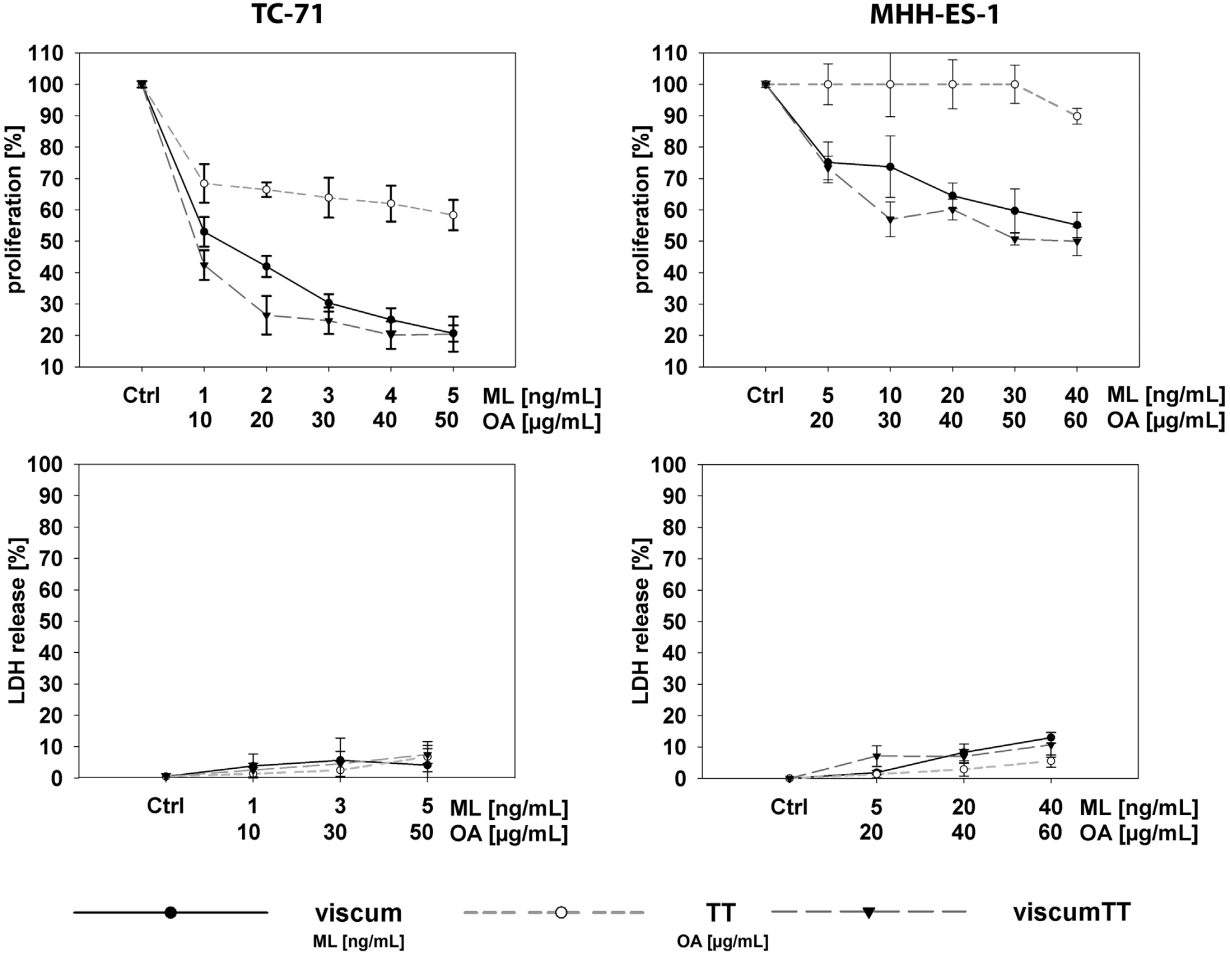
ViscumTT inhibits proliferation in Ewing sarcoma cell lines without early cytotoxicity. TC-71 and MHH-ES-1 cells were treated with increasing concentrations of TT, viscum or viscumTT for 24h and proliferation was estimated from total cell numbers in cultures started from defined cell numbers compared to control cultures (upper graphs). Cells were counted using a CASY^®^ Cell Counter. TC-71 and MHH-ES-1 cells were incubated with increasing concentrations of viscum, TT or viscumTT for 2h, before assessing early cytotoxicity via LDH release into the culture medium (lower graphs). All results are presented as the percentage of untreated control (Ctrl) cultures ± SD, and are the mean of 3 independent experiments. Mistletoe lectin (ML) and oleanolic acid (OA) concentrations were used as a measure of viscum and TT active agent extract concentration, respectively.

**Fig 2:**
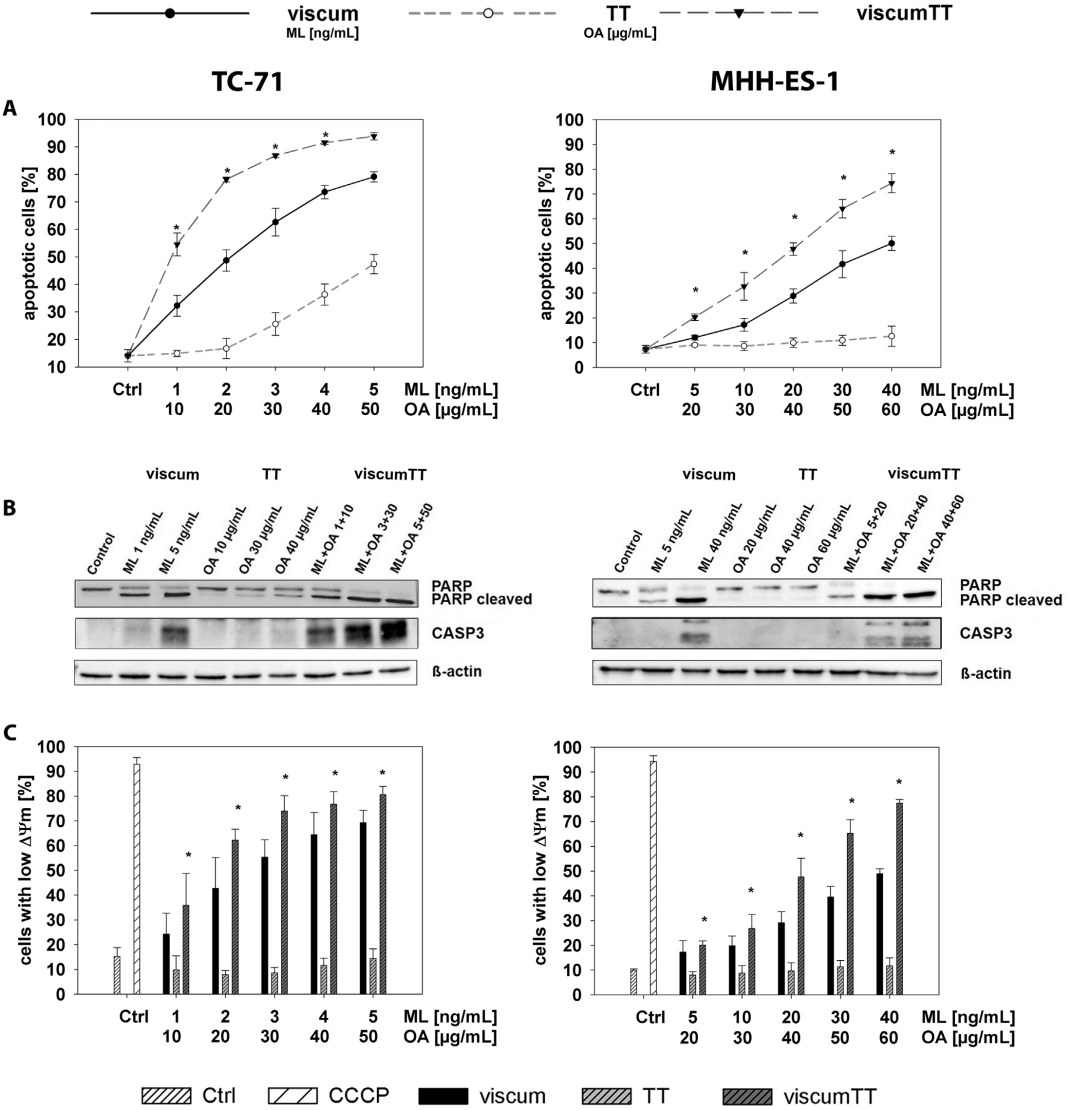
ViscumTT combined extract synergistically induces apoptosis in Ewing sarcoma cell lines. **A.** TC-71 and MHH-ES-1 cells were incubated for 24h with increasing concentrations of viscum, TT and viscumTT. Cultures were then stained with annexin V and propidium iodide and examined by flow cytometry. The percentage of dead cells in each treatment group are shown (±SD) from 3 independent experiments. A synergistic effect of the combined viscumTT extract above single extracts was calculated by Webb´s fractional product (*Fp > 1). **B.** PARP1 and CASP3 cleavage was assessed in whole-cell extracts from cells treated as described above using western blotting. β-actin was used as loading control, and images shown are representative for the results in 3 independent experiments. **C.** TC-71 and MHH-ES-1 cells treated as above for 24h and were assessed for mitochondrial involvement in apoptosis using JC-1 staining and flow cytometry. Bars depict the percentage of cells with low mitochondrial membrane potential (ΔΨ_m_, ± SD, n = 3) in each treatment group averaged from 3 independent experiments (±SD, error bars). A synergistic effect of combined viscumTT extracts was calculated by Webb´s fractional product (*Fp > 1). Carbonyl cyanide m-chlorophenyl hydrazine (CCCP) was used as positive control. Mistletoe lectin (ML) and oleanolic acid (OA) concentrations were used as a measure of viscum and TT active agent extract concentration, respectively.

We first assessed the cytotoxic potential of aqueous and triterpene components separately or in combination on Ewing cell lines *in vitro* to examine which extracts contained effectors from *Viscum album* L. and detect potential synergies. TC-71 and MHH-ES-1 cell proliferation was measured after incubation with increasing concentrations of viscum, TT or viscumTT. ViscumTT and viscum inhibited proliferation in a concentration-dependent manner in both cell lines (Fig 1 and S1 Table). TT had no effect on MHH-ES-1 cells, but displayed a low inhibitory effect in TC-71 cells (Fig 1 and S1 Table). To exclude the possibility that extracts caused early cytotoxicity via necrotic cell death, LDH release from cells into culture medium was measured after a 2h treatment pulse. Neither cell line released significant amounts of LDH in response to treatment with any extract (Fig 1 and S1 Table). We flow cytometrically assessed the fraction of TC-71 and MHH-ES-1 cells undergoing apoptosis in response to 24h incubation with *Viscum album* L. extracts after annexin V/propidium iodide staining. ViscumTT and viscum induced apoptosis in both cell lines in a dose-dependent manner, whereas TT only induced apoptosis in TC-71 cells (Fig 2A and S1 Table). Importantly, the reconstituted viscumTT extract displayed a synergistic effect on apoptosis in both cell lines (Fig 2A and S1 Table). Western blot analyses detecting PARP1 and CASP3 cleavage confirmed cells died from apoptotic induction (Fig 2B). Taken together, reconstituting *Viscum album* L. cytotoxic compounds in viscumTT had a synergistic and dose-dependent effect on proliferation and apoptosis in Ewing sarcoma cell lines.

### ViscumTT induces apoptosis via mitochondrial depolarization and activates caspases *in vitro*

**Fig 3:**
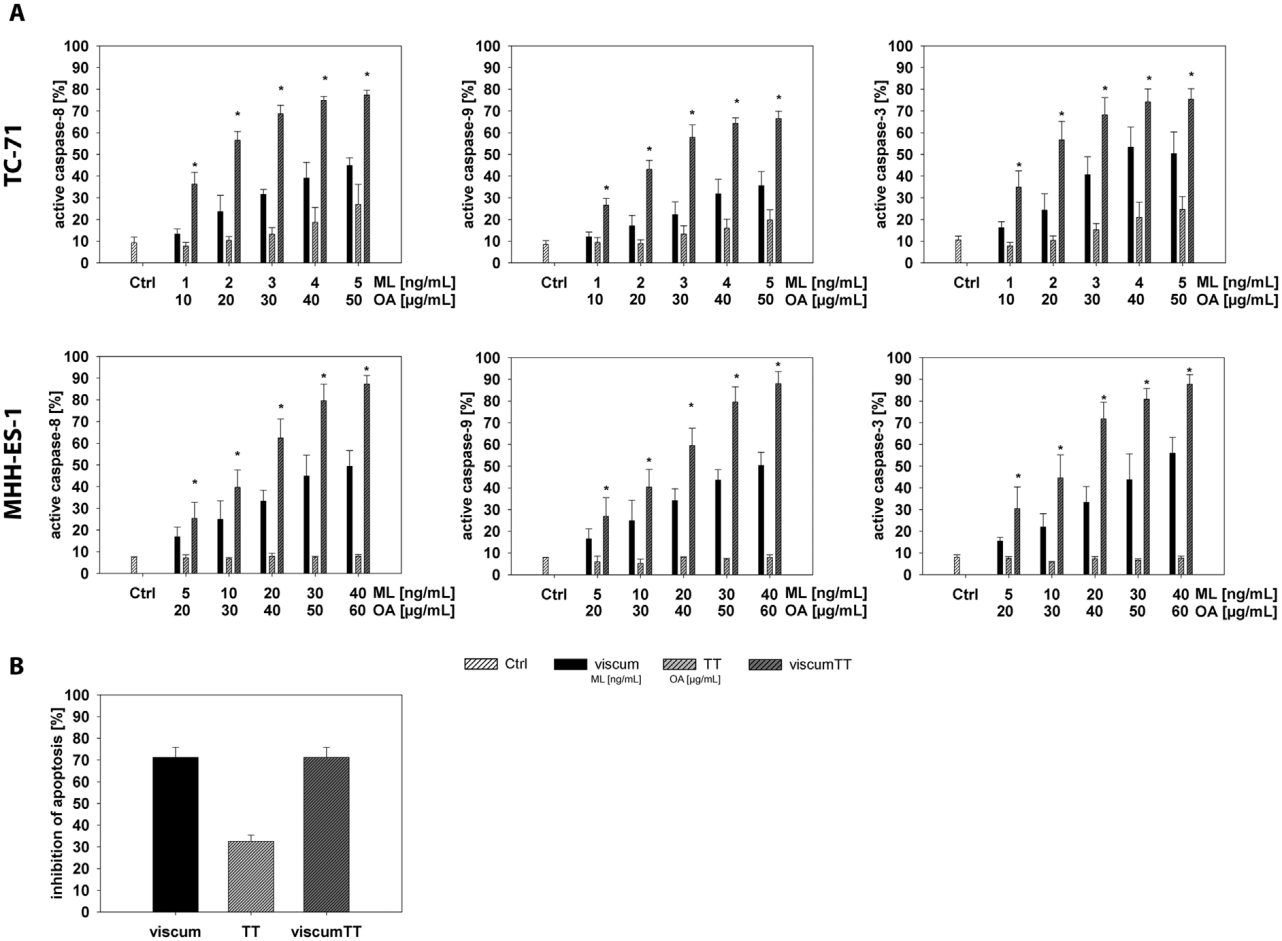
ViscumTT synergistically activates caspases in Ewing sarcoma cells lines above the levels activated by viscum alone. **A.** TC-71 and MHH-ES-1 cells treated 24h with either viscum, TT or viscumTT. CASP9, CASP8, CASP3 activation was measured using FITC-LEHD-FMK, FITC-IETD-FMK and FITC-DEVD-FMK staining followed by flow cytometry. Bars represent mean activation (±SD, error bars) in treatment groups from 3 independent experiments relative to untreated control cultures. A synergist effect of the combined viscumTT extracts was calculated by Webb´s fractional product (*Fp > 1). **B.** TC-71 cells were treated with viscum, TT and viscumTT for 24h with or without 40μM Z-VAD-FMK (pan-caspase inhibitor), then apoptotic cells were detected using annexin V/propidium iodide staining and flow cytometry. Bars represent the mean (±SD, error bars) of 3 independent experiments. Results are expressed as percentages of the untreated control cultures. Mistletoe lectin (ML) and oleanolic acid (OA) concentrations were used as a measure of viscum and TT active agent extract concentration, respectively.

To more closely assess the mechanism behind viscumTT cytotoxicity, we analyzed mitochondrial involvement and caspase activation during apoptotic induction in TC-71 and MHH-ES-1 cells, since most chemotherapeutic drugs induce mitochondrial membrane permeabilization as they induce apoptosis [35,36]. Cell cultures treated for 24h with viscum, TT or viscumTT extracts were flow cytometrically assessed after JC-1 staining to detect mitochondrial membrane potentials relative to untreated control cultures. Treatment with viscumTT or viscum alone resulted in a dose-dependent loss of mitochondrial membrane potential in TC-71 and MHH-ES-1 cells (Fig 2C and S1 Table). Similar to its effect on apoptosis, viscumTT synergistically affected mitochondrial membrane depolarization, peaking at an 80% suppression of membrane potential in both Ewing sarcoma cell lines (Fig 2C and S1 Table). TT treatment alone did not significantly affect mitochondrial membrane potential in either cell line (Fig 2C and S1 Table). We also measured the activation of CASP8, CASP9 and CASP3 in TC-71 and MHH-ES-1 cells following 24h treatment with viscum, TT or viscumTT. A similarly potent concentration-dependent caspase activation, reaching 80% in TC-71 and 90% in MHH-ES-1 cells, was observed in Ewing sarcoma cell lines after viscumTT or viscum treatment (Fig 3A and S1 Table). ViscumTT also synergistically increased caspase activation above the effects of viscum or TT extracts alone (Fig 3A and S1 Table). TT treatment, however, increased caspase activation by only ~15% in TC-71 cells and did not affect caspase activation in MHH-ES-1 cells (Fig 3A and S1 Table). These results suggest that in addition to the intrinsic pathway, the extrinsic apoptosis pathway also plays a role in viscum- and viscumTT-induced apoptosis. We also treated TC-71 cells for 24h with viscum, TT or viscumTT extracts in the presence or absence of the Z-VAD-FMK pan-caspase inhibitor. Z-VAD-FMK efficiently decreased apoptotic induction by up to 70% in cells treated with viscumTT or viscum and 30% in cells treated with TT (Fig 3B and S1 Table), validating the essential role of caspases in viscumTT- and viscum-induced apoptosis. These results reveal that *Viscum album* L. extracts induce apoptosis via the intrinsic (mitochondrial) and extrinsic apoptosis pathways, and that viscumTT synergistically reduces mitochondrial membrane polarization and caspase activation in Ewing sarcoma cells *in vitro*.

### Viscum, TT and viscumTT alter apoptosis-related protein expression

**Fig 4:**
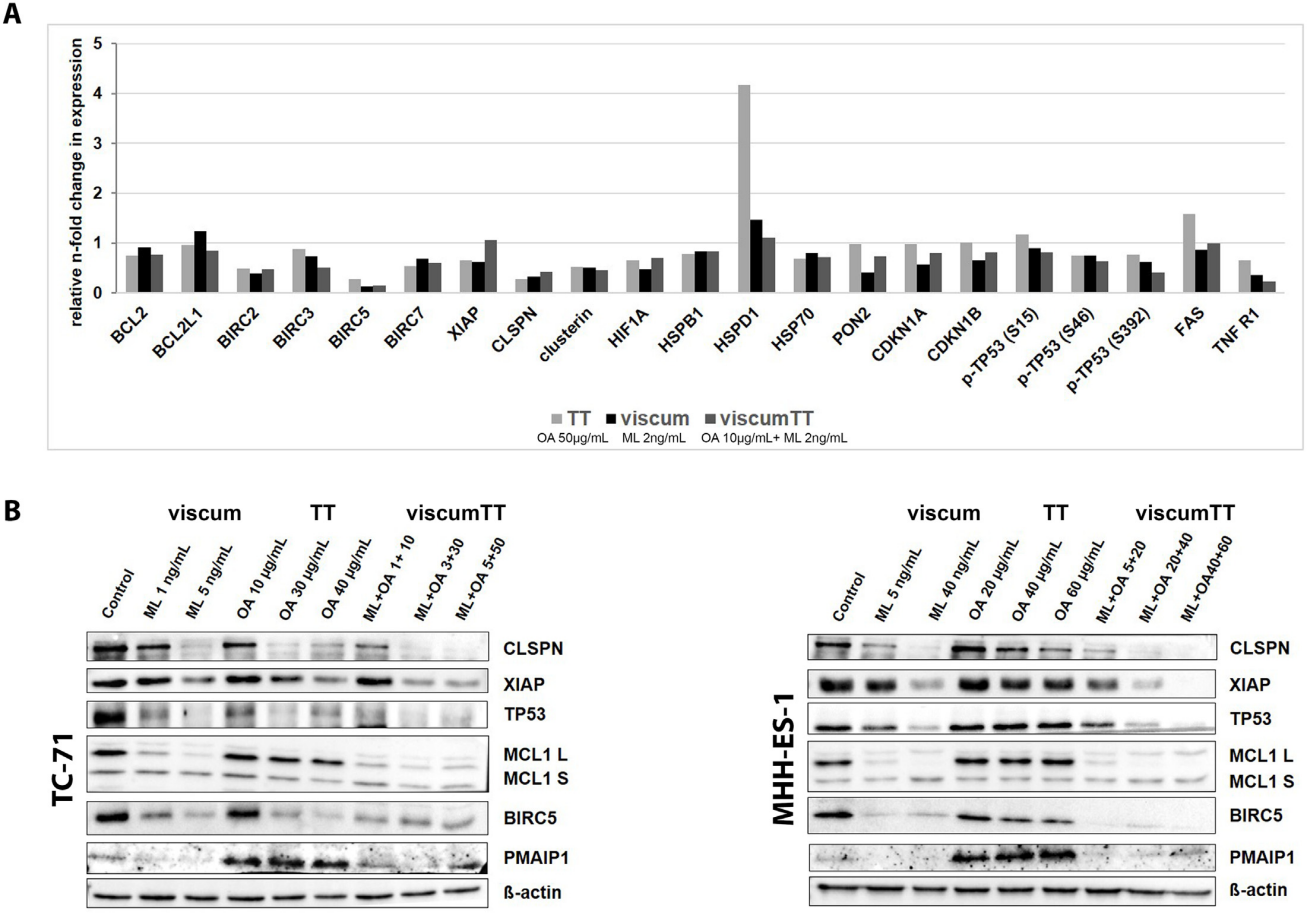
Viscum, TT and viscumTT alter apoptosis-related protein expression. **A.** Whole-cell protein extracts from TC-71 cells after 24h of treatment with viscum, TT or viscumTT concentrations approximating the IC50 were analyzed using the R&D systems human proteome profiler apoptosis array (n = 1). Bars represent the n-fold change in apoptosis-related protein expression relative to untreated control expression. **B.** TC-71 and MHH-ES-1 cells were treated with viscum, TT or viscumTT at the concentrations shown for 24h, then expression of the apoptosis-related proteins indicated was examined in whole-cell lysates using western blotting. Representative pictures are shown from 3 independent experiments. Mistletoe lectin (ML) and oleanolic acid (OA) concentrations were used as a measure of viscum and TT active agent extract concentration, respectively.

To more closely examine how mistletoe-derived cytotoxic agents induce apoptosis in Ewing sarcoma cells, we analyzed the expression of 35 proteins related to apoptosis using the R&D Systems human proteome profiler apoptosis array. TC-71 cells were treated with ~IC50 concentrations of viscum, TT or viscumTT. The array detected a shift of many proteins associated with apoptosis in protein extracts from the treatment groups. Viscum, TT and viscumTT reduced expression of the IAP family members, BIRC2 (cIAP1), BIRC3 (cIAP2), XIAP and BIRC5, and to a lesser extent, BCL2 protein and TP53, compared to untreated control cells (Fig 4A). BIRC5 downregulation was particularly prominent. Altogether, expression shifted the balance towards less anti-apoptotic protein expression. Interestingly, the expression of claspin (CLSPN), a protein needed for efficient DNA replication, was strongly suppressed by viscum, TT and viscumTT. TT also strongly upregulated expression of the heat shock 60kDa protein 1 (HSPD1). Since the array could only be performed on a single treatment experiment, we validated results in TC71 and MHH-ES-1 lysates from three independent experiments using western blotting. Western blots confirmed that viscum, TT and viscumTT downregulated BIRC5, XIAP, TP53 and CLSPN in Ewing sarcoma cells *in vitro* (Fig 4B). Since the pro-apoptotic BCL2 family member, PMAIP1 (NOXA), was shown to play a role in apoptosis induced by betulinic acid and doxorubicin polychemotherapy [37], we also analyzed PMAIP1 expression in extracts from cells treated with viscum, TT or viscumTT. TT treatment upregulated PMAIP1 expression in both cell lines, whereas viscum or viscumTT did not affect expression compared to the control (Fig 4B). ViscumTT and viscum, but not TT, effectively downregulated the anti-apoptotic protein, MCL1, in both cell lines compared to untreated control cells (Fig 4B). These data suggest that TT upregulates pro-apoptotic PMAIP1 but is incapable of downregulating MCL1, possibly explaining the less effective apoptotic induction of TT extract alone. Taken together, our results indicate that cytotoxic agents derived from mistletoe invoke several signaling pathways to induce apoptosis, which explains the synergistic effect of reconstituting a complete mistletoe extract in viscumTT.

### ViscumTT and viscum induce apoptosis and inhibit proliferation *ex vivo* and reduce xenograft tumor growth

**Fig 5:**
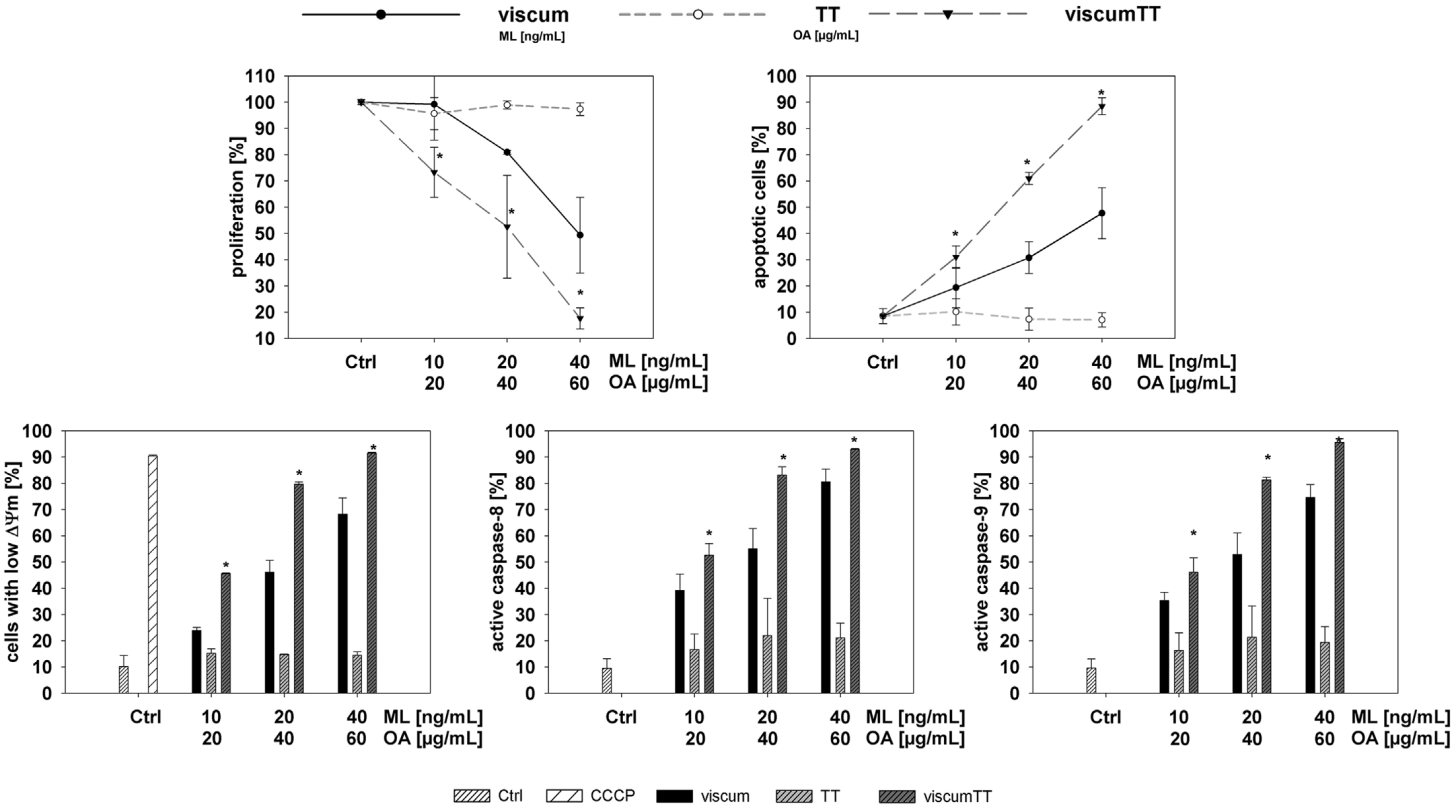
ViscumTT and viscum induce apoptosis and inhibit proliferation *ex vivo*. Primary Ewing sarcoma cells from a 15-year-old girl were grown as short-term cultures *ex vivo*, and treated with increasing concentrations of viscum, TT or viscumTT for 24h. Induction of apoptosis (n = 4), proliferation inhibition (n = 2), mitochondrial membrane potential (n = 2) and activation of CASP8 and CASP9 (n = 2) were measured flow cytometrically with the same assays described for the cell lines. Values are the means of n experiments ±SD (error bars). Controls were untreated cultures grown in parallel. A synergistic effect of the combined viscumTT extracts was calculated by Webb´s fractional product (*Fp > 1). Mistletoe lectin (ML) and oleanolic acid (OA) concentrations were used as a measure of viscum and TT active agent extract concentration, respectively.

**Fig 6:**
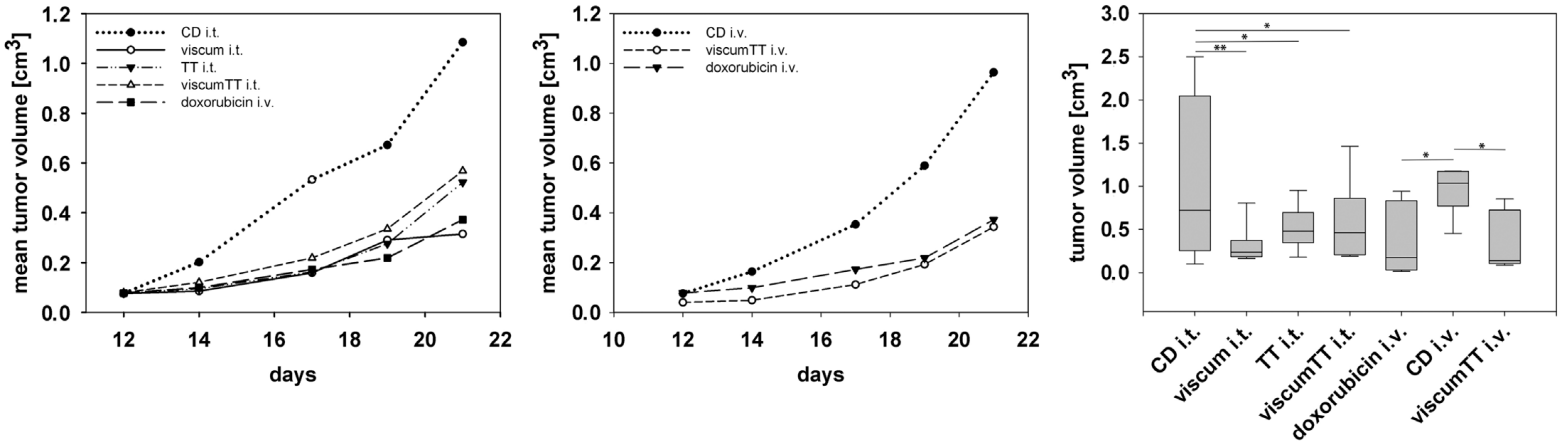
ViscumTT reduces the growth of human Ewing sarcoma xenograft tumors in mice. Established Ewing sarcoma xenograft tumors were intratumorally (i.t.) injected with 40/50/60mg oleanolic acid/kg mouse weight (TT i.t.), 0.75/1.25/1.75μg mistletoe lectin /kg mouse weight (viscum i.t.) or a combination thereof (viscumTT i.t.) every 2 to 3 days in increasing concentrations. Each concentration was administered twice within 2–3 days. One treatment group received viscumTT intravenously (i.v.), and one positive control group was once intravenously (i.v.) treated with 4 mg/kg doxorubicin. The negative control group received the solubilizing agent, cyclodextrins (CD), which was injected either intratumorally (CD i.t.) or intravenously (CD i.v.). One-way ANOVA revealed a significant group effect (p = 0.007) and Fisher´s Least Significant Difference test comparing all groups with all groups showed a significant effect between the CD i.t. control group and viscumTT i.t. (p = 0.033), viscum i.t. (p = 0.002) and TT i.t. (p = 0.021) as well as between the CD i.v. control group and viscumTT i.v. (p = 0.014) or the positive control group, doxorubicin i.v. (p = 0.015). Box-and-wisker plots each represent one treatment group including 8 mice (*p < 0.05, **p < 0.005).

**Fig 7:**
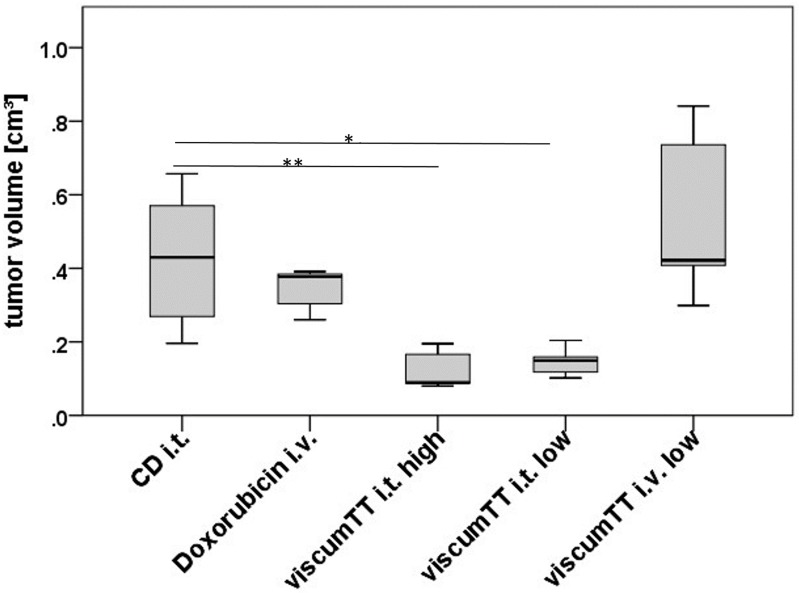
ViscumTT reduces the growth of patient-derived Ewing sarcoma xenograft tumors in mice. Established Ewing sarcoma xenograft tumors in NMRI-nu/nu mice were intratumorally (i.t.) and intravenously (i.v.) injected with 40/60/80mg/kg oleanolic acid and 1.25/1.75/2.25μg/kg mistletoe lectin in combination (viscumTT, low dose). Additionally, mice were treated intratumorally (i.t.) with 50/70/90mg/kg oleanolic acid and 1.25/1.75/2.25μg/kg mistletoe lectin in combination (viscumTT, high dose). Viscum TT was given every 2–3 days in increasing extract concentrations. One positive control group was treated twice intravenously (i.v.) with 4 mg/kg doxorubicin. The negative control group received cyclodextrins (CD) intratumorally (CD i.t.). One-way ANOVA revealed a strongly significant group effect (p < 0.0001) and Fisher´s Least Significant Difference test comparing all groups with all groups showed a significant effect between the CD i.t. control group and viscumTT i.t. low dose (p = 0.006) and viscumTT i.t. high dose (p = 0.003) Box-and-wisker plots each represent one treatment group including 5 mice (**p < 0.005).

We proceeded to verify our *in vitro* results in primary Ewing sarcoma cells grown *ex vivo* and in human Ewing sarcoma xenograft tumors grown in nude mice. *Ex vivo* cultures of primary Ewing sarcoma cells were treated with increasing concentrations of viscum, TT and viscumTT for 24h. TT alone had no effect on primary Ewing sarcoma cells *ex vivo* (Fig 5 and S1 Table), similarly to MHH-ES-1 cells. Treatment with viscum or viscumTT induced apoptosis in a concentration-dependent manner in primary Ewing sarcoma cells *ex vivo*, leading to mitochondrial membrane depolarization and CASP8 and CASP9 activation (Fig 5 and S1 Table). Importantly, we also validated the synergistic effect of viscumTT in primary Ewing sarcoma cells *ex vivo* (Fig 5 and S1 Table). We next tested the effect of viscum, TT and viscumTT on the established human Ewing sarcoma cell line TC-71 grown subcutaneously in female NOD/SCID IL2rγ null mice. Extracts were injected intratumorally every 2–3 days in increasing concentrations with each concentration being administered twice and a total of five treatments of TT, viscum or viscumTT (Fig 6). ViscumTT was also intravenously applied six times in one treatment group to assess systemic effects on tumor growth (Fig 6). We included a positive control group that received a single intravenous dose of the classical cytostatic agent, doxorubicin (Fig 6). The negative control group received the solubilizing agents, cyclodextrins, injected either intratumorally or intravenously (Fig 6). The administered extract concentrations were well-tolerated by mice, which displayed no significant weight loss or toxicity symptoms. All intratumorally injected *Viscum album* L. extracts significantly inhibited tumor growth by 50–70% (as assessed from final measurement of mean tumor volumes compared to controls intratumorally injected with cyclodextrins alone), with viscum most strongly inhibiting tumor growth (Fig 6). Intravenously administered viscumTT inhibited tumor growth by 65% (compared to controls receiving the cyclodextrin adjuvant intravenously, and achieved a comparable effect to doxorubicin, which reduced tumor growth by 62% (Fig 6). In addition, a patient derived xenograft was treated with viscumTT that was injected intratumorally (low and high dose) or intravenously (low dose) every 2–3 days in increasing concentrations and a total of nine treatments. We included a positive control group that received twice an intravenous dose of doxorubicin (Fig 7). The negative control group received cyclodextrins intratumorally (Fig 7). Again, the administered extract concentrations were well-tolerated by mice, which displayed no significant weight loss or toxicity symptoms. The intratumorally injected low and high dose of viscumTT significantly inhibited tumor growth by 66–71% (as assessed from final measurement of mean tumor volumes compared to controls intratumorally injected with cyclodextrins alone). By contrast, intravenously administered viscumTT and doxorubicin did not inhibit tumor growth significantly. Taken together, reconstituting whole mistletoe extract in viscumTT synergistically induced apoptosis in primary Ewing sarcoma cells *ex vivo* and effectively suppressed Ewing sarcoma growth in xenograft mouse models.

## Discussion

The combined mistletoe extract, viscumTT, inhibits proliferation and induces apoptosis in Ewing sarcoma cells *in vitro and ex vivo*, and efficiently reduces xenograft tumor volume *in vivo*. While viscum alone showed similar but less effective anticancer activity *in vitro* and *ex vivo*, the triterpene extract, TT, displayed significant activity only against TC-71 cells *in vitro* and TC-71 xenografts *in vivo*. Although it is ineffective against MHH-ES-1 and primary Ewing sarcoma cells, TT appears to potentiate the effect of viscum, creating the synergistic effect of viscumTT. Our data correlates with the well-known phytopharmacological phenomenon that a naturally existing combination is often more effective than the single compounds [38,39].

Our *in vitro* and *ex vivo* experiments showed viscumTT and viscum invoke both intrinsic and extrinsic pathways of apoptosis in Ewing sarcoma cells. Treatment of TC-71 cells with a pan-caspase inhibitor verified that apoptosis was caspase-dependent. This is in line with previous data from hepatocellular carcinoma and leukemia [30,40–42]. Notably, MHH-ES-1 cells and the primary cells were more resistant to mistletoe extracts than TC-71 cells, and required ~15-fold higher viscum concentrations to reach IC50. This demonstrates a variable responsiveness of different cell populations to viscum and viscumTT. Variable susceptibility could stem from different cell surface characteristics (glycocalyx), since mistletoe lectins specifically bind to D-galactose and/or N-acetyl-D-galactosamine [15] and therefore response to mistletoe lectins is cell-type-dependent [43]. TT proved less cytotoxic than either viscumTT or viscum against Ewing sarcoma cells. TT also did not activate CASP8, CASP9 or CASP3 or cause mitochondrial membrane depolarization, indicating other mechanisms cause the moderate TT-mediated apoptosis and proliferative inhibition in TC-71 cells. Triterpene acids, such as ursolic acid and oleanolic acid and its derivatives, have previously been shown to induce apoptosis in osteosarcoma, breast and pancreatic cancers via extrinsic or intrinsic pathways requiring caspases [25,44–48]. Oleanolic acid and its derivative have also been shown to induce apoptosis without caspase participation in pancreatic cancer cells and acute myelogenous leukemia [49,50], suggesting that the triterpene effect is cell type-dependent.

Our deeper assessment of the apoptotic mechanism after mistletoe extract treatment *in vitro* revealed altered expression of diverse apoptosis-related proteins shifting the balance towards apoptosis. Treatment with viscumTT and viscum effectively reduced expression of the anti-apoptotic protein, MCL1. The MCL1 downregulation in Ewing sarcoma cell lines agrees with previous data demonstrating MCL1 downregulation by mistletoe lectins in leukemia cells [51] and by an oleanolic acid derivative in osteosarcoma cells [52]. TT strongly upregulated the PMAIP1 pro-apoptotic protein in both Ewing sarcoma cell lines, but didn't affect MCL1 expression resulting in less effective apoptosis induction. PMAIP1 upregulation together with downregulation of the anti-apoptotic BCL2L1 protein have been correlated with increased apoptosis in pancreatic tumor cells treated with an oleanolic acid derivative [53]. Erhardt *et al*. reported that PMAIP1 is involved in apoptotic induction by drug combinations, such as doxorubicin and betulinic acid in diverse cancer cell lines, including breast, colon and leukemia [37]. ViscumTT contains oleanolic and betulinic acid, viscotoxins, mistletoe lectins and other undefined components, which together produce a phytopolychemotherapeutic effect. But unlike TT, viscumTT did not upregulate PMAIP1 expression in Ewing sarcoma cells, indicating PMAIP1 regulation is not involved in the synergistic effect produced by viscumTT. Mistletoe extracts also suppressed TP53 expression in Ewing sarcoma cell lines, but this effect is less meaningful since TC-71 expresses an inactive TP53 mutant protein [54] and MHH-ES-1 harbors an in-frame deletion of Ser215 in the TP53 transactivation domain. The downregulation might indicate signaling is in place that would trigger downregulation of the wild type tumor suppressor. However, this must not mean it would have an anti-apoptotic effect, since mistletoe lectins both suppressed wild type TP53 expression and induced apoptosis in human lymphocytes [55]. Other reports did not analyze TP53 expression directly, but also showed that *Viscum album* L. induced apoptosis in cell lines (SMMC7721 hepatocellular carcinoma and NALM-6 acute lymphoblastic leukemia cells) harboring wild type *TP53* [30,42]. CLSPN expression was also decreased in both cell lines after treatment with viscumTT or either single extract. It is required for efficient DNA replication during a normal S phase and has been shown to promote cancer cell survival [56]. CLSPN is an essential upstream regulator of checkpoint kinase 1, and triggers cell cycle arrest in response to replicative stress or DNA damage. In a previous study, we showed that mistletoe extracts similarly reduced CLSPN expression in acute myeloid leukemia cells, where extracts also induced apoptosis and inhibited proliferation [31]. Mistletoe extracts also reduced XIAP and BIRC5 expression in Ewing sarcoma cells. The IAP family regulates cell death by inhibiting caspases or the assembly of pro-apoptotic protein complexes, and mediating the expression of anti-apoptotic proteins [57,58]. Since BIRC5 expression is associated with chemo- and radioresistance in Ewing sarcoma and poor patient prognosis [9,10], BIRC5 downregulation by *Viscum album* L. extracts makes it an interesting candidate for targeting Ewing sarcoma. We previously achieved a similar effect with acute myeloid leukemia cells [31]. Oleanolic and ursolic acid have also been shown to induce apoptosis and downregulate XIAP in hepatocellular carcinoma cells [47], while oleanolic acid decreased BIRC5 expression in human nonsmall cell lung cancer and ovarian cancer cells [27,59] and betulinic acid derivatives downregulated BIRC5 in glioblastoma cell lines [60]. Taken together, our *in vitro* data shows that viscumTT combines the anti-cancer effects derived from triterpene acids and mistletoe lectins, and pro-apoptotically shifts the balance of apoptosis-related proteins, suggesting this effect is not cancer-type dependent.

The anticancer effects we observed for mistletoe extract *ex vivo* and *in vitro*, were confirmed *in vivo* in a TC-71 cell line and patient-derived xenograft model for Ewing sarcoma. In the TC-71 xenograft, all extracts inhibited the tumor growth significantly when injected intratumorally. Intravenous injection of viscumTT had a comparable effect on the tumor growth to doxorubicin treatment. However, doxorubicin was applied only once due to bad tolerability in mice. Still, this also emphasizes the good tolerability of the used mistletoe extracts. Noteworthy, we were not able to observe a synergistic effect of viscumTT in *vivo*. However, identical results *in vitro* and *in vivo* are impossible, since a living organism is more than one cell type and the extracts are metabolized in the body. Unfortunately, there is literature lacking showing the pharmacokinetics of mistletoe extracts in mice or humans, but Huber *et al*. showed for subcutaneous injections in humans, that serum mistletoe lectin levels are highly variable after injection [61]. Furthermore, Frantz *et al*. demonstrated, that mistletoe lectins can also bind Serum glycoproteins as haptoglobin, alpha 1-acid glycoprotein and transferrin inhibiting the cytotoxicity of the lectins [62] Although viscumTT had no synergistic effect *in vivo*, it still demonstrated effectiveness intratumorally and intravenously. To validate the effect of viscumTT *in vivo*, we injected viscumTT in a patient-derived xenograft model intratumorally and intravenously. The intratumorally injected viscumTT in low and high dose significantly reduced tumor growth, while twice application of doxorubicin showed no significant tumor reduction. Notably, viscumTT intratumoral displayed different results depending on the used mouse stem demonstrating the difficulties to evaluate *in vitro* results *in vivo*. Mistletoe lectins have also been reported to have an anticancer effect in mouse models for melanoma, pre-B ALL and lymphosarcoma [63–65], as were oleanolic acid and its derivatives in mouse models of melanoma, osteosarcoma and lung cancer [27,66,67]. We and others have previously demonstrated the *in vivo* efficacy of viscumTT against mouse models of acute lymphoblastic leukemia and melanoma [30,33].

Here we demonstrate that reconstituting the aqueous and triterpene components from mistletoe in the viscumTT extract synergistically induces caspase-dependent apoptosis associated with CLSPN, MCL1, BIRC5 and XIAP downregulation in Ewing sarcoma cell lines *in vitro* and primary cells *ex vivo*. ViscumTT also reduced tumor growth in TC-71- derived and patient-derived Ewing sarcoma xenografts. ViscumTT may represent a phytopolychemotherapeutic option as a promising adjuvant therapy for patients with Ewing sarcoma.

## Supporting Information

S1 Table All *in vitro* and *ex vivo* measurements. TC-71 cells, MHH-ES-1 cells and Ewing sarcoma primary cells were incubated for 24 h with the extracts in depicted concentrations followed by measurements of apoptosis, proliferation, mitochondria membrane potential (JC-1), active caspases, LDH.
(XLSX)

## Abbreviations

IAP: inhibitor of apoptosis protein
ML: mistletoe lectin
TT: triterpene acid-containing extract
LDH: lactate dehydrogenase
TBST: Tris-buffered saline with Tween-20
